# Autochthonous Outbreak of SARS-CoV-2 Omicron Variant in Booster-Vaccinated (3 Doses) Healthcare Workers in Southern Italy: Just the Tip of the Iceberg?

**DOI:** 10.3390/vaccines10020283

**Published:** 2022-02-13

**Authors:** Daniela Loconsole, Lucia Bisceglia, Francesca Centrone, Anna Sallustio, Marisa Accogli, Lidia Dalfino, Nicola Brienza, Maria Chironna

**Affiliations:** 1 Department of Interdisciplinary Medicine, University of Bari, 70124 Bari, Italy; daniela.loconsole@uniba.it (D.L.); nicola.brienza@uniba.it (N.B.); 2 Strategic Regional Health and Social Agency of Puglia (AReSS Puglia), 70121 Bari, Italy; l.bisceglia@aress.regione.puglia.it; 3 Hygiene Section, Department of Biomedical Sciences and Human Oncology, University of Bari, 70124 Bari, Italy; francesca.centrone@uniba.it (F.C.); marisa.accogli@uniba.it (M.A.); 4 Hygiene Unit, Azienda Ospedaliero-Universitaria Consorziale Policlinico di Bari, 70124 Bari, Italy; annasallustio@libero.it; 5 Anesthesia and Intensive Care Unit, Azienda Ospedaliero-Universitaria Consorziale Policlinico di Bari, 70124 Bari, Italy; lidia.dalfino@policlinico.ba.it

**Keywords:** SARS-CoV-2 Omicron variant, outbreak, COVID-19 vaccine, surveillance, epidemiology, hospitalization, Italy

## Abstract

The Omicron variant of concern (VOC), first detected in Italy at the end of November 2021, has since spread rapidly, despite high vaccine coverage in the Italian population, especially in healthcare workers (HCWs). This study describes an outbreak of SARS-CoV-2 Omicron infection in 15 booster-vaccinated HCWs. On 16 December 2021, two HCWs working in the same ward were infected with SARS-CoV-2. The Omicron VOC was suspected due to S gene target failure on molecular testing. Further investigation revealed that 15 (65%) of 23 HCWs attending a social gathering on 13 December were infected with Omicron, as shown by whole-genome sequencing, with a phylogenetic tree suggesting a common source of exposure. Five of these HCWs experienced mild symptoms. A patient with multiple chronic conditions hospitalized in the same ward was also infected by one of the HCWs involved in the outbreak. Despite being booster vaccinated, this patient required ICU treatment. Ten subjects achieved negativity in 10–19 days. The outbreak in booster-vaccinated subjects confirms the high transmissibility and immune evasion of the Omicron VOC. More stringent non-pharmaceutical interventions, administration of booster doses, and genomic surveillance are crucial long-term strategies to mitigate the consequences of the spread of the Omicron VOC.

## 1. Introduction

On 14 November 2021, a new variant of SARS-CoV-2 virus was identified in an immunocompromised patient in South Africa [1]. Subsequently, the case rates in Gauteng province of South Africa increased faster than in previous waves [2], with this new variant overtaking Delta as the predominant lineage in South Africa [3]. The new SARS-CoV-2 strain belongs to the B.1.1.529 lineage and is characterized by an unusually high number of mutations in the spike protein. Some of these mutations are of particular concern, as they could be associated with immune escape potential and higher transmissibility [1]. The B.1.1.529 variant was designated a variant of concern (VOC) by the World Health Organization on 26 November 2021 and was given the name Omicron [3]. One of the characteristic mutations of the Omicron VOC is the 69/70 deletion in the spike protein, which impairs the detection of the S gene (S gene target failure [SGTF]) using common commercial real-time PCR kits [3]. The same mutation was present in the Alpha VOC, the spread of which was monitored using the SGTF approach [4,5,6]. Therefore, similar to the Alpha SARS-CoV-2 variant, the SGTF can be considered a useful proxy marker for the early identification and monitoring of the spread of this new VOC [1,7].

The immunity conferred by vaccination is likely to be lower in subjects infected with the Omicron than with the Delta VOC [8]. Moreover, preliminary evidence suggests an increased risk of reinfection with this variant [1]. However, people who either are booster-vaccinated or who were previously infected are likely to have stronger protection against Omicron [8].

As of 20 December 2021, Omicron has been detected in most countries, and the number of COVID-19 cases caused by this variant is increasing exponentially worldwide [8]. The overall number of confirmed cases of Omicron infections in the European Union and European Economic Area (EU/EEA) by 19 December 2021 was 4691, with most being autochthonous cases [9]. The rapid spread of the Omicron VOC has led to the expectation that this variant will become the predominant one in a short time and that Christmas holiday gatherings may further accelerate this trend [8].

Regarding the disease, more data are needed to understand the severity profile of the Omicron VOC. Preliminary data on characteristics and outcomes of subjects involved in the fourth wave in South Africa showed that, compared with the previous SARS-CoV-2 waves, fewer patients presenting to the Emergency Department were admitted to the hospital and that, among hospitalized patients, the degree of disease severity and mortality rates were lower [10]. These features may be associated with the more limited capacity the variant to replicate in lung tissue than in bronchial tissue [11]. Moreover, the relative low hospitalization rates due to infection with Omicron in South Africa may be associated with the high levels of previous infection [12].

In Italy, the first cases of Omicron VOC were identified at the end of November 2021 [13]. Following the identification of these cases and in accordance with European guidelines [14], the Italian Ministry of Health has endeavored to enhance the genomic surveillance of SARS-CoV-2 strains. This may allow early identification of cases of Omicron infection, enabling the application of public health measures to contain the spread of the Omicron VOC at the national level [15]. The most recent national survey in Italy performed to estimate the prevalence of VOCs showed a very rapid increase in the prevalence of the Omicron VOC, increasing from 0.19% [16] to 80.75% of cases in less than 1 month [17]. In the Apulia region, the first two cases of Omicron VOC were identified in samples collected on 13 December 2021 [13].

The present study describes an autochthonous outbreak of SARS-CoV-2 Omicron VOC in healthcare workers (HCWs) vaccinated with three doses of BNT162b2 mRNA COVID-19 vaccine (booster-vaccinated) in the Apulia region of Southern Italy, which occurred after they attended a social gathering in early December 2021. The outbreak was investigated in depth to increase knowledge about the Omicron variant and to develop better targeted infection control measures.

## 2. Patients and Methods

On 16 December 2021, following molecular tests that showed positivity for SARS-CoV-2 and SGTF, two HCWs working in the same ward were suspected of being infected with SARS-CoV-2 Omicron VOC. An outbreak investigation to trace other probable cases identified 15 HCWs infected with SARS-CoV-2 Omicron VOC. The clinical presentation of cases was classified according to the National Institute of Health (NIH) clinical staging of COVID-19 disease [18]. Nasopharyngeal swabs (UTM, FLOQ Swabs TM, Copan Italia, Brescia, Italy) were processed at the Laboratory of Molecular Epidemiology and Public Health of the Hygiene Unit (A.O.U.C. Policlinico Bari, Italy), the coordinator of the Regional Laboratory Network for SARS-CoV-2 diagnosis in the Apulia region. RNA was extracted using MagMAX Viral/Pathogen NucleicAcid Isolation Kits (Thermo Fisher Scientific, Waltham, MA, USA), and molecular testing was performed using TaqPath RT-PCR COVID-19 Assays, a three-target commercial multiplex real-time PCR assay based on the identification of the N, ORF1ab, and S genes (Thermo Fisher Scientific). SGTF-positive samples were screened for the presence of notable types of spike protein mutations (HV 69–70 deletion, N501Y, K417N, E484K, and K417T) using commercial multiplex real-time PCR kits (Seegene Allplex SARS-CoV-2 Variants I and II Assay, Arrows Diagnostics, Genova, Italy). For each positive sample, the cycle threshold (Ct) values were recorded for all three genes as an indirect index of viral load and a valuable proxy for infectious virus [19,20]. Additionally, whole-genome sequencing (WGS) was performed using the Ion Torrent platform (ThermoFisher Scientific, Waltham, MA, USA), as previously described [21]. Phylogenetic analysis was performed using the Nextclade sequence analysis webapp (https://clades.nextstrain.org/, accessed on 14 February 2021). Strains closely related to those identified in the outbreak were evaluated by analysis of strains in the GISAID database [13] (www.gisaid.org, accessed on 11 January 2021). Clade information was described using the GISAID and Nextstrain nomenclature, and lineage information was described using the Pangolin nomenclature. The sequences of 10 strains detected during the outbreak were deposited in the GenBank database (accession numbers: EPI_ISL_8143019, EPI_ISL_8142277, EPI_ISL_8140764, EPI_ISL_8925453, EPI_ISL_8925543, EPI_ISL_8925551, EPI_ISL_8925610, EPI_ISL_8925645, EPI_ISL_8925646, EPI_ISL_8926378).

## 3. Results

On 15 December 2021, two HCWs working in the surgery ward of the Policlinico Hospital of Bari, Apulia region, presented with cough and cold. Nasopharyngeal swabs taken from these two HCWs on 16 December 2021 were positive for SARS-CoV-2. Because multiplex real-time PCR showed SGTF in both HCWs and according to the definition of the Italian Ministry of Health [15], these two subjects were regarded as probably infected with the Omicron VOC. Epidemiological investigation revealed that these two subjects had attended a Christmas dinner with their colleagues on 13 December 2021. Twenty-three attendees, all HCWs, were identified and nasopharyngeal swabs were obtained. The 23 subjects included six (26%) men and 17 (74%) women, of average age 32 years. Overall, 15 (65%) HCWs tested positive for SARS-CoV-2 (Table 1), with all 15 positive samples showing an SGTF. Molecular screening for variant identification revealed the presence of the del69/70, N501Y, and K417N mutations.

**Figure 1:**
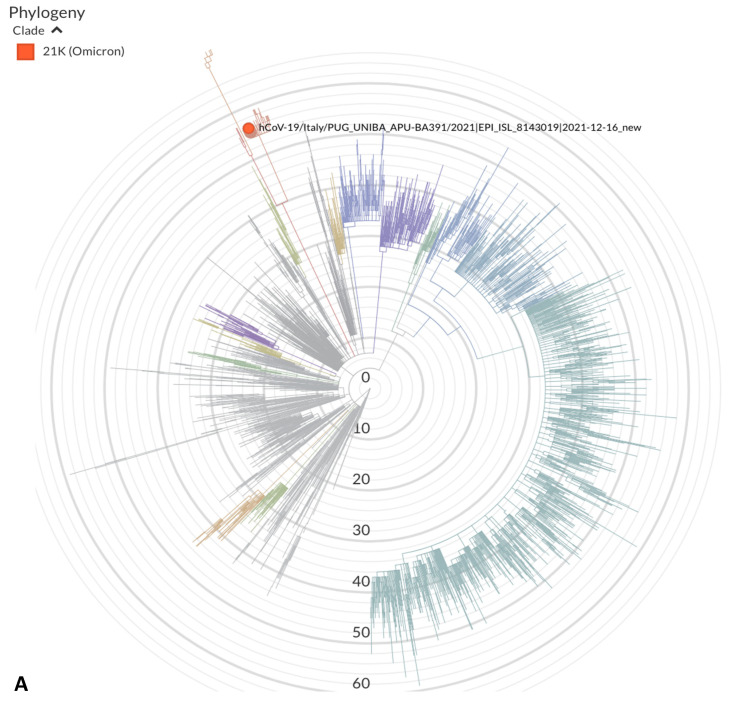

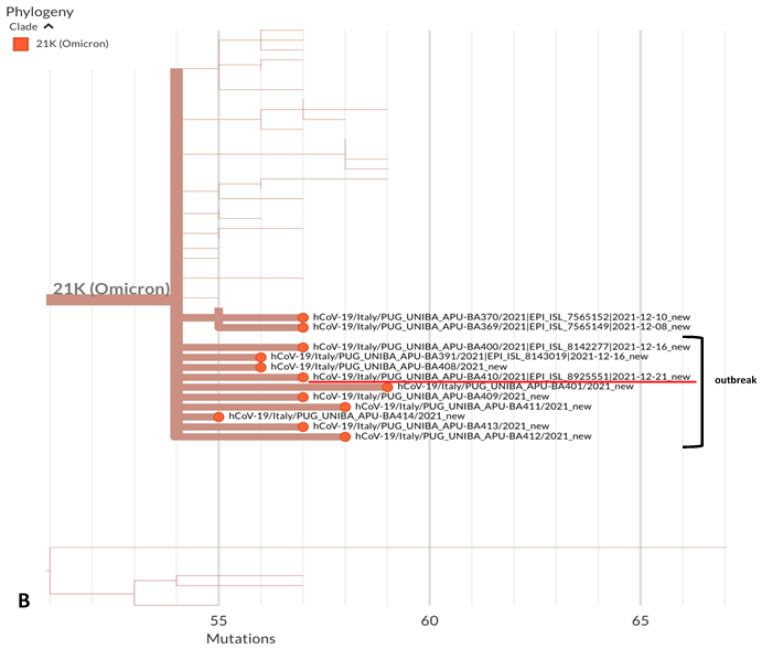
(**A**) Phylogenetic analysis of hCoV19/Italy/PUG_UNIBA_APU_BA391/2021. Other genomes were retrieved from GISAID (https://www.gisaid.org, accessed on 14 February 2021). (**B**) Phylogenetic tree of 12 severe acute respiratory syndrome coronavirus 2 (SARS-CoV-2) Omicron VOC full genome sequences from Apulia, including the 10 genomes identified in this study. Clade information using the GISAID and Nextstrain nomenclatures is shown. The strain of the patient is underlined in red.

The Ct values of all positive samples were low, averaging 21. Of the 15 positive subjects, five showed mild symptoms, and the other 10 were asymptomatic. All of the positive HCWs were booster-vaccinated. WGS of nine strains showed that they were closely related to each other, confirming a common source of exposure (Figure 1). Sequences of five strains were not successfully obtained. 

The HCWs negative for SARS-CoV-2 were not quarantined, but they were required to wear ffp2 masks during working shifts and to monitor themselves for fever or other symptoms consistent with COVID-19. Nasopharyngeal swabs taken 7 and 10 days after exposure were all negative for SARS-CoV-2. 

On the basis of the suspicion of a cluster caused by the Omicron VOC, all the 25 patients hospitalized in the involved ward were screened on 17 December 2021 and found to be negative for SARS-CoV-2 infection. No patients were exposed but discharged before the screening date was identified. On 21 December, a 49-year-old woman hospitalized for a surgical aortic and mitral valve replacement, which had been performed on 15 December, showed respiratory distress and required intensive care support. The patient was affected by kidney failure requiring dialysis, high blood pressure, and monoclonal gammopathy of undetermined significance. A molecular test performed on 21 December 2021 showed that she was positive for SARS-CoV-2. The patient had been vaccinated with three doses of the BNT162b2 vaccine, the third vaccination on 21 September 2021. WGS showed that the strain detected in this patient was closely related to those identified in the HCWs involved in the outbreak, confirming nosocomial infection (Figure 1). 

The clinical conditions of the patient improved, and she was transferred to the Pneumology ward on 27 December 2021. Because these HCWs worked in a non-COVID-19 ward, they wore surgical masks during their working shifts.

At the time of this writing, molecular testing showed that 11 of these subjects had become negative for SARS-CoV-2, whereas five subjects remained positive. The median time from diagnosis to negativity was 14 days (range: 10–19 days). All symptomatic subjects recovered after few days. The patient who required intensive care support recovered completely and was discharged on 4 January 2022. 

## 4. Discussion

The outbreak described in this study underlines some important issues about the effectiveness of vaccines against the transmission of the Omicron VOC and the need to strengthen non-pharmaceutical interventions (NPIs) to contain its spread, especially in HCWs. These findings may also provide insight into the natural history of SARS-CoV-2 Omicron VOC infections. 

The cluster described here could not be linked to previous cases of Omicron VOC infection. Thus, despite travel restrictions and extended contact tracing efforts [22], the Omicron VOC showed widespread community transmission beginning in early December 2021. An ongoing community transmission of Omicron VOC was also reported in Denmark [23], with the earliest cases of Omicron in Denmark identified in samples collected before the first announcement of this variant from South Africa [23].

The subjects involved in the outbreak were all booster-vaccinated. Currently available evidence shows that the efficacy of currently available vaccines is significantly reduced against the Omicron VOC [14,24], as well as against symptomatic infection [25]. Of the cluster of 15 HCWs, five (33%) showed mild symptoms and none required hospitalization. By contrast, the patient who had already been hospitalized for chronic clinical conditions showed severe symptoms. Initial data from the United Kingdom have shown that one dose of any vaccine shows a VE against hospitalization of 52%, that two doses are associated with vaccine effectiveness (VE) rates of 72% for up to 24 weeks after the second dose and 52% after 25 weeks [25], and that three doses result in a VE rate of 88% [25]. In a study performed in South Africa, however, the overall estimated VE rate after two doses of the BNT162b2 vaccine against hospitalizations caused by the Omicron VOC was 70%, lower than the 93% estimated for the Delta variant [26]. The addition of a booster dose has been reported to improve VE rates against severe outcomes [27,28]. Despite all subjects in this outbreak having been vaccinated, they became infected, suggesting that the mRNA vaccines have low rates protection against the Omicron VOC. In addition, prolonged viral shedding was observed, confirming that mucosal immunity was poorly activated by mRNA vaccines, thus failing to limit virus entry through this route [29]. However, clinical outcomes in all of these subjects were considered favorable.

The attack rate reported for the outbreak in Bari was 65%, similar to that reported for an outbreak caused by the Omicron VOC in early December 2021 in booster-vaccinated HCWs in the Faroe Islands [30], but lower than that reported for an outbreak in Norway in November 2021 [7]. None of the subjects in the latter study, however, had received the booster dose, suggesting that three doses of vaccine could reduce the transmissibility of the Omicron VOC, thereby improving the VE.

The incubation period for infections caused by the Omicron VOC was shorter than that for infections caused by the Delta VOC [24]. Moreover, the doubling time for the Omicron VOC was short [3,31]. The median time between infection and the onset of symptoms in the infected HCWs current study was 2.5 days, similar to that in an Omicron VOC cluster in Nebraska in November–December 2021 [24]. The latter outbreak was also characterized by five cases of reinfection of vaccinated people, suggesting waning immunity and/or partial immune evasion by Omicron [24].

The most worrisome aspect of the outbreak described in this study was the transmission of the SARS-CoV-2 Omicron VOC from an HCW involved in the outbreak to a patient hospitalized for diseases other than COVID-19. This finding confirms that vaccinated subjects can be infectious and can transmit SARS-CoV-2 virus despite the booster dose. Non-COVID-19 hospital areas should therefore be considered high-risk environments for SARS-CoV-2 outbreaks. The virus may originate from patients or HCWs who interact with others in environments inside and outside the hospital, and may especially affect vulnerable patients who are hospitalized [32]. Wearing surgical masks, according to protocols adopted in non-COVID-19 wards, may be insufficient to completely avoid SARS-CoV-2 dissemination in hospital wards, especially because the Omicron VOC is highly transmissible. Therefore, wearing ffp2 masks by HCWs is now advisable. However, since only one patient in 25 was infected, wearing a surgical mask could be also considered useful. Compared with the general population, HCWs are at higher risk of repeated exposure to SARS-CoV-2 infection in hospital settings and, despite the use of personal protective equipment such as ffp2 masks, face shields, double gloves, and gowns, reinfection and nosocomial outbreaks caused by wild-type and Alpha strains of SARS-CoV-2 have occurred [20,21]. Because Omicron VOC is three- to sixfold more infectious than previous variants [33], measures of infection control in non-COVID-19 wards require strengthening. 

As suggested by the European Centre for Disease Prevention and Control [34], NPIs that were used to control previous variants of SARS-CoV-2 should be further strengthened in response to the ongoing spread of the Omicron VOC. Future goals should include modifying or improving current vaccines to mitigate the spread of the Omicron VOC. 

## 5. Conclusions

The Omicron VOC is characterized by very high transmissibility and a high capacity to cause outbreaks, even among booster-vaccinated subjects such as HCWs. This VOC can also cause severe disease in subjects with comorbidities. Timely reporting of the spread of this new variant is crucial, as is the importance of avoiding nonessential travel and social activities, particularly during the period of the year characterized by Christmas gatherings. Delays in implementing interventions to contain the spread of SARS-CoV-2 could result in rapid dissemination of the virus, stress on healthcare systems, and deaths [35]. Even if the severity of infections caused by the Omicron VOC is lower than that reported for the Delta VOC [10], the exponential growth of cases associated with the increased transmissibility of the Omicron VOC could rapidly outweigh any benefits of potentially reduced severity. 

Finally, the rapid increase of cases caused by superspreading events has challenged the mitigation measures that have been introduced to contain the further spread of SARS-CoV-2 [23]. Vaccines are a crucial long-term strategy to mitigate the consequences of the spread of SARS-CoV-2. Utmost priority should be given to vaccinating people not yet vaccinated or not fully vaccinated. The administration of booster doses could accelerate the control of the virus and its process towards endemicity.

## Figures and Tables

**Table 1:** Demographic, clinical, laboratory, and vaccination data of the healthcare workers (HCWs) and the patient involved in this outbreak.

Case	Sex	Age (Years)	Clinical Status	Symptom Onset	Date of Sample Collection	Date of Negative Sample	Real-Time PCR (Ct *)			Vaccine (BNT162b2 mRNA COVID-19 Vaccine)		
							N Gene	ORF1ab Gene	S Gene	Dose I	Dose II	Dose III
1 (HCW)	F	29	Mild	15/12/2021	16/12/2021	-	20	21	-	07/01/2021	29/01/2021	26/10/2021
2 (HCW)	F	40	Asymptomatic		16/12/2021	04/01/2022	19	20	-	11/01/2021	03/02/2021	02/11/2021
3 (HCW)	F	31	Asymptomatic		18/12/2021	29/12/2021	26	27	-	07/01/2021	29/01/2021	19/11/2021
4 (HCW)	F	32	Asymptomatic		16/12/2021	04/01/2022	14	15	-	07/01/2021	29/01/2021	27/10/2021
5 (HCW)	F	43	Asymptomatic		17/12/2021	-	15	16	-	11/01/2021	03/02/2021	25/10/2021
6 (HCW)	F	32	Mild	15/12/2021	17/12/2021	04/01/2022	20	20	-	11/01/2021	03/02/2021	06/12/2021
7 (HCW)	M	29	Asymptomatic		20/12/2021	-	15	16	-	12/01/2021	04/02/2021	16/11/2021
8 (HCW)	M	30	Asymptomatic		17/12/2021	04/01/2022	29	29	-	09/02/2021	03/03/2021	11/11/2021
9 (HCW)	M	29	Asymptomatic		20/12/2021	30/12/2021	25	25	-	11/01/2021	03/02/2021	03/11/2021
10 (HCW)	F	29	Mild	16/12/2021	17/12/2021	31/12/2021	20	21	-	05/01/2021	27/01/2021	22/10/2021
11 (HCW)	F	29	Mild	18/12/2021	17/12/2021	-	18	19	-	07/01/2021	29/01/2021	28/10/2021
12 (HCW)	M	41	Asymptomatic		16/12/2021	04/01/2022	17	17	-	12/01/2021	04/02/2021	03/11/2021
13 (HCW)	M	25	Asymptomatic		16/12/2021	04/01/2022	31	31	-	05/01/2021	27/01/2021	03/11/2021
14 (HCW)	F	28	Mild	15/12/2021	16/12/2021	30/12/2021	26	28	-	05/01/2021	27/01/2021	14/10/2021
15 (HCW)	F	29	Asymptomatic		20/12/2021	-	20	20	-	09/01/2021	01/02/2021	03/11/2021
16 (Patient)	F	49	Severe	21/12/2021	21/12/2021	03/01/2022	26	26	-	30/03/2021	20/04/2021	21/09/2021

* Ct = cycle threshold; F = female; M = male.

## Data Availability

All data are available from the corresponding author by e-mail request.
